# High-Risk ExPEC from Commensal Phylogroup A: Genomic Characterization of a Bovine Meningoencephalitis Isolate, BN01

**DOI:** 10.3390/microorganisms14071586

**Published:** 2026-07-21

**Authors:** Jingjing Ren, He Qin, Wenliang Yan, Yayin Qi, Dongdong Du, Pengyan Wang, Wenli Yan, Jianjun Jiang

**Affiliations:** 1 College of Animal Science and Technology, Shihezi University, Shihezi 832000, China; renjingjing@shzu.edu.cn (J.R.); qinhe_621@163.com (H.Q.); yayinqi@shzu.edu.cn (Y.Q.); wwpy_322@163.com (P.W.); 2 Xinjiang Production and Construction Corps Animal Husbandry and Veterinary Work Station, Urumqi 830017, China; wenliang0034@163.com; 3 Analysis and Testing Center, Xinjiang Academy of Agriculture and Reclamation Science, Shihezi 832000, China; dudongdong1201@163.com; 4 College of Food Science and Technology, Shihezi University, Shihezi 832000, China

**Keywords:** extraintestinal pathogenic *Escherichia coli*, bovine, neonatal meningitis, comparative genomics, antimicrobial resistance genes, *cdiA*, One Health

## Abstract

Extraintestinal pathogenic *Escherichia coli* (ExPEC) causes severe infections in humans and animals, yet bovine isolates remain poorly characterized. Here, we report the first complete genome of a bovine ExPEC strain, BN01 (serotype O101:H9-ST10-phylogroup A), isolated from calf meningoencephalitis. Unlike classical ExPEC that typically belong to B2/D phylogroups and O1/O2/O18 serotypes, BN01 represents the A-ST10-O101 sublineage that has emerged as predominant among bovine ExPEC populations. The genome comprises a chromosome encoding 197 virulence factors, with *cdiA* uniquely identified in BN01 compared to six other representative ExPEC genomes—a contact-dependent growth inhibition system, and three distinct plasmids: a conjugative ESBL carrier (*bla*CTX-M-164, IncI1), a bovine-associated multidrug resistance island (IncY), and a mobilization-ready vector (IncFII). Animal virulence assays demonstrated that intraperitoneal challenge with *E. coli* BN01 caused 80% mortality (8/10 mice) within the observation period and yielded an LD_50_ of 10^6.3^ CFU/mouse. These findings demonstrate that high-risk ExPEC can occur in phylogroups typically associated with commensal strains, expanding the conventional understanding of phylogroup–virulence associations. They highlight the need to assess the zoonotic potential of livestock-associated atypical lineages and support integrated genomic surveillance under the One Health framework.

## 1. Introduction

Pathogenic *Escherichia coli* can be classified into intestinal pathogenic *E. coli* (IPEC) and extraintestinal pathogenic *E. coli* (ExPEC), both of which cause a wide range of infectious diseases in humans and animals. ExPEC strains include newborn meningitis-causing *E. coli* (NMEC) and uropathogenic *E. coli* (UPEC) as major pathotypes [1]. With high pathogenicity and mortality, ExPEC is recognized as a globally important pathogen with zoonotic potential. Infections caused by ExPEC not only pose a serious threat to public health but also impose a substantial economic burden [2].

The virulence mechanisms of ExPEC have been extensively studied. ExPEC employs a coordinated arsenal of virulence factors to overcome host defenses, including adhesins for colonization, invasins for cellular entry, iron acquisition systems, serum resistance factors, and secretion systems that deliver effector molecules [3]. Meanwhile, the spread of antimicrobial resistance (AMR) genes and multidrug-resistant (MDR) bacteria across humans, animals, and the environment presents a major challenge under the One Health framework [4]. Plasmids play a central role in the ecology and evolution of high-risk ExPEC clones, serving as key vehicles for the horizontal transfer of resistance and virulence determinants across diverse hosts and environments [5]. Widespread antibiotic use has exacerbated the MDR phenotype in ExPEC, and MDR ExPEC strains are now frequently reported in clinical settings worldwide, complicating treatment. Of particular concern is the potential transmission of resistance genes from animal-derived ExPEC to human pathogens through multiple routes, including the food chain and direct contact with animals or contaminated environments, underscoring the importance of farm-to-fork surveillance under the One Health framework [6].

Despite the clinical importance of ExPEC, genomic studies have predominantly focused on isolates from humans, poultry, and swine [7,8,9], leaving bovine ExPEC strains largely uncharacterized at the genomic level. Recent molecular epidemiological studies have identified the A-ST10-O101 sublineage as a predominant clone among bovine ExPEC populations in China [10], yet complete genomic characterization of this emerging lineage remains unavailable. Moreover, with the rapid expansion of cattle farming, ExPEC-associated infections in cattle—such as neonatal meningitis and septicemia—are increasingly reported, raising concerns about potential zoonotic transmission and the spread of resistance determinants. Therefore, comprehensive genomic characterization of bovine ExPEC strains—particularly the emerging A-ST10-O101 lineage—is urgently needed to elucidate their pathogenic potential and AMR profiles.

In this study, we characterized *E. coli* BN01, a clinical ExPEC strain (serotype O101:H9) isolated in our previous work from the brain and liver of a calf with meningoencephalitis during an acute outbreak in Shihezi, Northwest China. BN01 belongs to the A-ST10-O101 sublineage, and this study provides the first complete genome for a bovine isolate of this lineage. Through murine meningitis experiments and complete genome sequencing, we aim to elucidate its pathogenic and resistance mechanisms, contributing to genomic surveillance of emerging bovine ExPEC under the One Health framework.

## 2. Materials and Methods

### 2.1. Statement on Experimental Animals and Ethics

This study was conducted in strict accordance with experimental animal ethics guidelines and utilized 6- to 8-week-old SPF BALB/c mice obtained from the Experimental Animal Center of Shihezi University. Among them, 20 mice were used for survival rate analysis, 60 for median lethal dose (LD_50_) determination, and 16 for infection model establishment, with gender balance maintained across all experimental groups. All procedures were approved by the Animal Experiment Ethics Committee of Shihezi University (Approval No.: A2022-019). Experimental animals were housed in individually ventilated cage (IVC) systems under constant environmental parameters (temperature 22 ± 1 °C, humidity 55 ± 5%, 12 h light/dark cycle), with free access to sterilized feed and ultraviolet-treated drinking water. Health status was monitored daily by professional veterinarians. Preemptive analgesia was administered throughout the experiment. Animals were euthanized using carbon dioxide followed by cervical dislocation, and tissue samples were systematically collected for secondary analysis, thereby comprehensively upholding animal welfare standards and ensuring compliance with research ethics.

### 2.2. Murine Infection Model and Virulence Assessment

Survival rate analysis: Twenty mice were randomly divided into two groups (*n* = 10 per group). The experimental group received an intraperitoneal (*i.p.*) injection of 0.3 mL bacterial suspension at 1.4 × 10^7^ CFU/mL, while the control group received an equal volume of phosphate-buffered saline (PBS). Mortality was recorded daily for 14 days, and survival curves were plotted.

Median lethal dose (LD_50_) determination: Sixty mice were randomly assigned to five experimental subgroups (*n* = 10 per subgroup) and one control group (*n* = 10). Bacterial suspensions were serially diluted and then administered *i.p.* at a volume of 0.3 mL. The control group received PBS. Mice were observed for 14 days, and the LD_50_ was calculated using the Kerber method [11].

Infection model: Sixteen mice were randomly divided into infection (*n* = 8) and control (*n* = 8) groups using a random number table, with group allocation concealed in sealed envelopes. The infection group received *i.p*. injection of BN01 suspension at a dose equivalent to 2/3 LD_50_, while the control group received PBS. Clinical symptoms were monitored daily for 7 days. At 72 h post-infection, mice were euthanized, and brain tissues were collected for bacterial recovery and histopathological analysis. Bacterial identification was performed by 16S rDNA PCR (forward primer: 5′-TACAGGTGACTGCGGGCTTATC-3′; reverse primer: 5′-CTTACCGGGCAATACACTCACTA-3′). The reaction conditions were as follows: pre-denaturation at 94 °C for 5 min; 30 cycles of denaturation at 94 °C for 45 s, annealing at 59 °C for 30 s, and extension at 72 °C for 30 s; and a final extension at 72 °C for 10 min. Culturing was conducted on eosin methylene blue (EMB) agar. Brain tissue sections were stained with hematoxylin and eosin (H&E) for histopathological examination.

### 2.3. Genomic Sequencing and Assembly

Genomic DNA of *E. coli* BN01 was extracted and sequenced by Novogene (Beijing, China). A SMRTbell library was prepared using the PacBio Sequel II platform. In brief, high-quality DNA was sheared using a Covaris g-TUBE, followed by DNA damage repair, end polishing, and adapter ligation. The library was purified with AMpure PB magnetic beads and quantified using a Qubit fluorometer (Pacific Biosciences, Menlo Park, CA, USA), with fragment size distribution assessed on an Agilent 2100 Bioanalyzer (Agilent Technologies, Santa Clara, CA, USA). Raw sequencing reads were filtered to obtain high-quality clean data. De novo genome assembly was performed using SMRT Link v5.1.0. Plasmid maps were visualized with the BLAST Ring Image Generator (BRIG) v0.95, and resistance gene structures were illustrated using SnapGene v3.3.1.

### 2.4. Genome Annotation and Functional Analysis

Protein-coding genes were predicted using GeneMarkS (v4.17) with default parameters. tRNAs were identified with tRNAscan-SE (v1.3.1) (E-value ≤ 1 × 10^−5^, covariance model score ≥ 20), rRNAs with Barrnap (v0.9) (E-value < 1 × 10^−5^, coverage ≥ 0.8), and sRNAs with CMsearch (Infernal v1.1rc4) using Rfam covariance models (E-value ≤ 1 × 10^−2^). Prophages were predicted with PhiSpy (v2.3). Genomic islands (GIs) were identified using IslandPath-DIMOB (v0.2) based on phylogenetic bias and detection of mobile genetic elements (e.g., transposase/integrase genes).

Functional annotation was performed by searching all predicted open reading frames (ORFs) against the following databases using DIAMOND (v2.0.15) (E-value ≤ 1 × 10^−5^, identity ≥ 40%, coverage ≥ 40%): Gene Ontology (GO, accessed May 2022), Kyoto Encyclopedia of Genes and Genomes (KEGG, release 2022-05-01), Clusters of Orthologous Groups (COG, version 2020), Non-Redundant Protein Database (NR, accessed May 2022), Transporter Classification Database (TCDB, version 9.4.0.1), Pfam (version 35.0), Swiss-Prot (UniProt release 2022_01), and Carbohydrate-Active Enzymes (CAZy, accessed May 2022). Plasmid homology and synteny analysis were visualized using the BLAST Ring Image Generator (BRIG) v0.95. Genetic structures of antimicrobial resistance genes and their surrounding regions were generated using SnapGene v3.3.1.

### 2.5. Virulence and Resistance Gene Analysis

Virulence genes were identified by aligning amino acid sequences against the Virulence Factor Database (VFDB, version 2022.01) using DIAMOND (identity ≥ 40%, coverage ≥ 70%). Antimicrobial resistance genes were identified using the Comprehensive Antibiotic Resistance Database (CARD, version 3.2.3) with the same alignment criteria.

### 2.6. Comparative Genomics and Phylogenetic Analysis

For comparative genomic analysis, seven representative *E. coli* strains were selected, encompassing three distinct pathogenic and commensal categories. These included one phylogroup A commensal strain, *E. coli* K-12 MG1655 (NC_000913), serving as a reference for commensal background. The remaining six strains represented classical ExPEC lineages associated with human and animal extraintestinal infections: the APEC strain APECO1 (NC_008563.1); the UPEC strain UMN026 (NC_011751); three NMEC strains, S88 (NC_011742), CE10 (NC_017646) and IHE3034 (NC_017628); and the porcine ExPEC strain PCN033 (CP006632). Collectively, this panel captures the diversity of ExPEC genotypes commonly implicated in extraintestinal infections across different hosts.

Phylogenetic reconstruction was performed using RAxML (v7.0.4) based on a core genome single-nucleotide polymorphism (SNP) matrix, employing the general time-reversible (GTR+Γ) model with 1000 bootstrap replicates. Genome-wide average nucleotide identity (ANI) was calculated using FastANI (v1.33), with a species delineation threshold of ≥95%. MLST was performed in silico using the *E. coli* MLST database. ST was determined from the seven standard housekeeping gene loci.

### 2.7. Antimicrobial Susceptibility Testing

Antimicrobial susceptibility was determined using the Kirby–Bauer disk diffusion method following Clinical and Laboratory Standards Institute (CLSI) M100-Ed33 and European Committee on Antimicrobial Susceptibility Testing (EUCAST) v13.0 guidelines. The following antimicrobial disks (Hangzhou Microbiological Reagent Factory, Hangzhou, China) were used at the indicated concentrations: amikacin (30 µg), gentamicin (10 µg), tobramycin (10 µg), ampicillin (10 µg), cefotaxime (30 µg), cefazolin (30 µg), ceftazidime (30 µg), amoxicillin-clavulanate (20/10 µg), doxycycline (30 µg), minocycline (30 µg), tetracycline (30 µg), chloramphenicol (30 µg), ciprofloxacin (5 µg), imipenem (10 µg), ertapenem (10 µg), and co-trimoxazole (1.25/23.75 µg). A 0.5 McFarland bacterial suspension was inoculated onto Mueller–Hinton agar plates. Antibiotic disks at standard concentrations for Enterobacteriaceae were applied. After incubation at 35 ± 1 °C for 16–18 h, inhibition zone diameters were measured and interpreted according to CLSI breakpoints. *E. coli* ATCC 25922 was used as the quality control strain.

## 3. Results

### 3.1. Survival Rate Analysis and LD_50_ Determination in Mouse Model

Following inoculation with BN01, mice in the experimental group exhibited progressive clinical signs of infection. Within 12 h post-infection, animals displayed lethargy, huddling behavior, and reduced feed and water consumption. After 24 h, mortality onset was observed, with necropsy revealing hepatosplenomegaly, intestinal mucosal shedding, and focal intestinal congestion with hemorrhage. Survivors exhibited emaciation and ruffled fur. The PBS control group remained asymptomatic throughout the 14-day observation period, with no gross pathological lesions at necropsy.

Survival analysis demonstrated 80% mortality (8/10) in the BN01-infected group, whereas the PBS group achieved 100% survival (Figure 1). LD50 determination using the Karber method yielded a value of 10 ^6.3^ CFU/mouse (Table 1).

### 3.2. Isolation and Identification of Bacteria from Mouse Brain Tissue

Brain tissue smears were stained with Wright’s stain, revealing small, blue, rod-shaped bacilli (indicated by arrows, Figure 2A). Following inoculation onto eosin methylene blue agar and incubation at 37 °C for 18–24 h, colonies displayed a metallic green sheen (Figure 2B). Gram staining of isolated colonies showed small, pink, rod-shaped bacilli (Figure 2C). The 16S rDNA gene sequence was amplified by PCR, yielding a band of approximately 622 bp (Figure 2D). These findings confirmed successful recovery of BN01 from the brain tissues of infected mice.

### 3.3. Histopathological Observation of Mouse Brain Tissue

Histopathological examination of brain tissue sections from BN01-infected mice revealed the following findings: In the blank control group, brain tissue architecture appeared normal without significant pathological alterations (Figure 3A). Pathological changes in the experimental group included dilated and congested submeningeal vessels (Figure 3B) and parenchymal and submeningeal vascular congestion with microthrombi (Figure 3C). The interstitium exhibited diffusely distributed vacuoles of varying sizes (Figure 3D), with concurrent intracytoplasmic vacuolation observed in cells (Figure 3E). Additionally, perivascular spaces were notably widened (Figure 3F). Characteristic neurological signs and gross pathological lesions (e.g., meningeal hemorrhage), and histopathological findings in brain tissues from the original bovine case from which the BN01 isolate was obtained are detailed in Supplementary Figure S1.

### 3.4. General Features of E. coli BN01

The complete genome sequence of *E. coli* BN01 was deposited in the NCBI database under accession numbers CP187563 (chromosome), CP187564 (plasmid 1, designated Plas1), CP187565 (plasmid 2, Plas2), and CP187566 (plasmid 3, Plas3). BN01 was identified as serotype O101:H9 (sequence type ST10, phylogroup A). The genome comprised a 4.96 Mb circular chromosome with a GC content of 50.7%, and three plasmids: Plas1 (IncI1, 110.4 kb, 49.62% GC), Plas2 (IncY, 73.6 kb, 52.87% GC), and Plas3 (IncFII, 77.6 kb, 51.82% GC) (Figure 4; Table 2). A total of 22 rRNAs and 87 tRNAs were annotated on the chromosome.

Gene islands (GIs) were predicted using IslandPath-DIMOB. No GIs were identified on the three plasmids. On the chromosome, 20 GIs were identified, of which 18 exceeded 10 kb in size (Figure 5). Detailed characteristics of each GI are provided in Table S1. Several GIs carried genes associated with antimicrobial resistance and virulence. GI5 contained a multidrug resistance island, including the aminoglycoside resistance genes *aph(6)-Id* and *aph(3″)-Ib*, the chloramphenicol resistance gene *floR*, the tetracycline resistance genes *tet(A)* and *tetR(A)*, and the macrolide resistance gene *mph(A)*. Additionally, a class 1 integron and transposase genes were identified within GI5. GI6 harbored the aminoglycoside resistance gene *aac(3)-IId* and mobile genetic elements, including a DNA invertase, transposase, and site-specific recombinase.

GI19 contained multiple recombinases/integrases, suggesting horizontal acquisition, as well as virulence-associated adhesins, including a fimbrial adhesin and a hemagglutinin. Furthermore, GI3 and GI14 encoded the type III and type VI secretion systems, respectively.

### 3.5. Comparison of E. coli BN01 with Other ExPEC

To determine the phylogenetic position of the bovine-derived strain BN01, we performed comparative genomic analysis with seven representative ExPEC strains from human, poultry, and porcine hosts. ANI analysis confirmed that BN01 belongs to the same species as all reference strains, with pairwise identities consistently exceeding the 95% species threshold (Figure 6). Core-genome phylogeny revealed that BN01 clusters with the laboratory strain *E. coli* MG1655 with high bootstrap support, and this clade is sister to a lineage comprising the porcine ExPEC strain *E. coli* PCN033 and the human urinary tract infection strain *E. coli* UMN026 (Figure 7; Table 3). Strikingly, despite this close genetic relatedness to MG1655, BN01 exhibits a serotype and phylogroup profile (O101:H9, phylogroup A) that diverges markedly from the classic O1/O2/O18 lineage typically associated with human and avian ExPEC. This phylogenetic position suggests that BN01 belongs to an atypical ExPEC sub-lineage with affinity to porcine isolates, distinguishing it from major ExPEC clades commonly found in humans and other hosts.

### 3.6. Functional Annotation of the E. coli BN01 Genome

Protein-coding sequences were annotated against multiple databases, including GO, KEGG, COG, NR, TCDB, CAZy, and PHI (E-value ≤ 1 × 10^−5^). BN01 possessed the core metabolic framework typical of *E. coli*, with enrichment in carbohydrate and amino acid metabolism pathways (Figures S2–S4). Furthermore, 73.2% of its genes showed high homology to *E. coli* (Figure S5). A total of 979 membrane transport proteins and 118 carbohydrate-active enzymes were identified (Figures S6 and S7), suggesting robust environmental adaptation capacity. Notably, 291 pathogen–host interaction genes were annotated from the PHI database, including 22 virulence-enhanced mutants (Figure S8).

### 3.7. Virulence Factor Analysis

Virulence factor analysis revealed that BN01 carries a total of 197 virulence genes, of which 192 were located on the chromosome and five on plasmid 1 (Table S2). Plasmid 1 harbored five adhesion-associated genes—*pilQ*, *pilR*, *pilS*, *pilV*, and *pilW*—all of which are involved in type IV pilus assembly. The chromosome contained numerous virulence-related genes, including those involved in pilus formation and adhesion (ecpA/B/C/D/E/R, *elfA*/*B*/*C*/*D*/*E*/*R*, *eaeH*); adhesion *(cgs*, *ecp*, *hep*, *fim*, *pil*); invasion *(ibes*, *cheB*/*R*/*W*/*Y*/*Z*, *motA*, *tia*); iron acquisition (*iroN*); transport (*cdiA*/*B*, *cah*, *ehaA*, *upaG*); and toxin production (*hlyA*/*E*).

Although BN01 was phylogenetically positioned as sister to the porcine/human UTI clade comprising PCN033 and UMN026 (Figure 7), it was isolated from a case of calf encephalitis, prompting a comparison of its virulence gene repertoire with those of other meningitis-associated strains (CE10, S88, and IHE3034). A Venn diagram revealed 147 virulence factors shared among the four genomes, with BN01 uniquely possessing 11 specific virulence factors (Figure 8). These 11 genes were identified as *agn43*, *cdiA*, *cdiB*, *espL4*, *espR4*, *espX5*, *espY1*, *spaQ*, *stjC*, *wzb*, and *wzi*. Further comparison of BN01 with PCN033, APECO1, UMN026, and the non-pathogenic strain MG1655 showed that *cdiA* was exclusively present in BN01 and absent from the other four genomes.

### 3.8. Drug Resistance Analysis

Antimicrobial resistance genes were identified using the CARD. A total of 63 resistance genes were detected in the BN01 genome, of which 55 were located on the chromosome, two (*bla*CTX-M-55 and *bla*CTX-M-164) on Plas1, and six (*bla*TEM-1, *aph(6)-Id*, *aph(3″)-Ib*, *sul2*, *dfrA14*, and *tet(A)*) on Plas2. No resistance genes were identified on Plas3.

These resistance genes confer resistance through four major mechanisms: antibiotic efflux, antibiotic inactivation, target alteration, and reduced permeability (Table 4). Among them, 36 genes (59%) were associated with antibiotic efflux, primarily involving ABC-type, RND-type, and MFS-type efflux pumps. Additionally, 14 genes were involved in antibiotic inactivation, eight in target alteration, and three in reduced permeability.

Consistent with the genomic prediction, antimicrobial susceptibility testing confirmed that BN01 exhibited resistance to multiple antibiotic classes, with particularly strong resistance to β-lactams, while remaining susceptible to polypeptide antibiotics (Table S3).

### 3.9. Plasmid Biology and Horizontal Transfer Potential

#### 3.9.1. Comparative Genomics and Global Homology of BN01 Plasmids

Plas1 (IncI1) exhibited extensive homology to plasmids from diverse hosts and geographic regions (Figure 9A). It showed >95% nucleotide identity to plasmids from *E. coli*, *Salmonella enterica*, *Shigella sonnei*, and *Klebsiella pneumoniae* isolated from human clinical samples (blood, stool, urine) and poultry in China, Japan, Belgium, and Korea.

Plas2 (IncY) shared backbone homology with pF16EC0456-1 (human blood, Korea) and RHB41-E2-C01 (swine farm deposits, UK) (Figure 9B). The ~30 kb resistance island harboring six ARGs (*tet(A)*, *bla*TEM-1, *aph(6)-Id*, *aph(3″)-Id*, *sul2*, and *dfrA14)* was present in the bovine isolate BN01 but absent from these matching human and swine IncY plasmids, indicating host-restricted acquisition of multidrug resistance.

Plas3 (IncFII, 77.6 kb) showed homology to IncFII plasmids from *E. coli* in the USA and Japan (Figure 9C). Notably, Plas3 carried no antimicrobial resistance genes.

#### 3.9.2. Plasmid Architectures and Resistance Gene Landscapes

Linear annotation revealed distinct functional strategies underlying the three plasmids’ adaptive roles (Figure 10).

Plas1 (110.4 kb) displayed a modular IncI1-1α architecture. The backbone contained replication (*repA_1*), maintenance (*parM_1*, *umuC_1*, *xerC_1*), and conjugative transfer machinery (*traC_1* encoding a type IV secretion system component). Plas1 carried a single ARG, *bla*CTX-M-164. This gene was located adjacent to two insertion sequences, IS91 and IS1380, both situated immediately downstream of *bla*CTX-M-164.

Plas2 (73.6 kb) harbored a densely IS-populated resistance island. The ~30 kb region exhibited modular IS architecture: *tet(A)-Tn3*, *bla_TEM-1-IS607*, a zero-spacer *aph(6)-Id–aph(3″)-Ib–sul2* cluster (4.1 kb), and *IS6-dfrA14.* The accumulation of IS6/IS110/Tn3 family elements indicates high genetic plasticity and stepwise assembly via multiple transposition events. The IncY backbone carried toxin–antitoxin systems (*relE*, *higB*) and type IV secretion genes (virB family), ensuring stable maintenance and conjugative mobility.

Plas3 (77.6 kb) was ARG-negative but retained a complete F-type conjugative apparatus. *traI* (relaxase), *traD* (coupling protein), *traM*/*traY* (transfer regulation), *finO* (fertility inhibition), and *traA*/*traV*/*traN* (pilus assembly). An IS5/IS903 element was detected, representing a “standing genetic variation” architecture—intact transfer machinery without resistance cargo.

## 4. Discussion

### 4.1. Genomic Basis of Enhanced Virulence in a Bovine NMEC Strain

The bovine NMEC strain BN01, isolated from a calf with fatal meningoencephalitis, exhibited high virulence in a murine meningitis model (LD_50_ 10^6.3^ CFU/mouse). Its complete genome (4.96 Mb chromosome; serotype O101:H9, ST10, phylogroup A; three plasmids IncI1, IncY, and IncFII) provides a framework for understanding its pathogenic and resistance traits, which surpasses that reported for several other ExPEC strains under comparable conditions [12,13].

BN01 harbors diverse adhesins, invasins, iron acquisition systems, and secretion systems. Of particular interest are the type I fimbrial genes *fimH* and *fimC*, as *FimH* mediates adhesion to brain microvascular endothelial cells (BMECs)—a critical step in the pathogenesis of *E. coli* meningitis [14,15]. The presence of invasion-associated genes *ibeB*, *ibeC*, and *hek*—with IbeB being essential for BMEC invasion and blood–brain barrier traversal [16,17]—further supports the neuropathogenic potential of BN01.

A distinctive feature of BN01 is the presence of *cdiA*, encoding a contact-dependent inhibition (CDI) system protein. CDI systems enhance bacterial competitiveness and have been linked to biofilm formation and virulence in uropathogenic *E. coli* [18,19]. Notably, *cdiA* was absent from the six other representative ExPEC genomes analyzed in this study, suggesting that this gene may confer a competitive advantage in polymicrobial environments—a trait potentially relevant to bovine host colonization.

### 4.2. Genomic Determinants of Antimicrobial Resistance and the Role of Mobile Genetic Elements

Antimicrobial susceptibility testing confirmed that BN01 is resistant to multiple antibiotic classes (β-lactams, aminoglycosides, tetracyclines, sulfonamides, and macrolides), consistent with the 63 resistance genes identified in its genome. While many resistance genes are chromosomal, key determinants—including the ESBL gene blaCTX-M-164 and a six-gene multidrug resistance island—reside on two conjugative plasmids, underscoring the role of mobile genetic elements in resistance dissemination.

The three plasmids in BN01 exhibit a striking functional complementarity. Plas1 (Incl1) carries *bla*CTX-M-164 within a complex IS91/IS1380 environment. Homology analysis reveals close relationships to plasmids from human clinical isolates and poultry across multiple countries, suggesting inter-species dissemination potential [20,21]. Plas2 (IncY, 73.6 kb) harbors a unique ~30 kb multidrug resistance island containing six resistance genes [*aph(6)-Id*, *aph(3″)-Ib*, *sul2*, *tet(A)*, *bla*TEM-1, *dfrA14*] embedded in a modular IS architecture. BLASTn analysis against the NCBI database indicates that while closely related IncY backbone sequences exist in human and swine isolates, none carry this specific resistance island configuration. This absence strongly suggests that this island is unique to this bovine ExPEC strain and may reflect host-specific adaptive evolution driven by antimicrobial selection pressure in cattle farming environments [22,23]. Plas3 (IncFII, 77.6 kb) lacks resistance genes but retains complete F-type conjugative machinery (*traI*/*D*/*M*/*Y*/*O*/*A*/*V*/*N*). IncFII plasmids typically encode virulence or resistance determinants that drive clinical dissemination [24,25], whereas Plas3 retains transfer capacity without resistance cargo—representing a “transfer-proficient but resistance-naïve” scaffold.

In addition to plasmids, genomic islands (GIs) contribute to the resistance repertoire. GI5 harbors a multidrug resistance region including *aph(6)-Id*, *aph(3″)-Ib*, *floR*, *tet(A)*, and *mph(A)*, along with a class 1 integron; GI6 carries the aminoglycoside resistance gene *aac(3)-IId*. The presence of class 1 integrons—key players in resistance gene capture and expression in Gram-negatives [26,27]—within these GIs suggests mobilization potential. The co-occurrence of three conjugative plasmids with distinct incompatibility groups and complementary IS repertoires, together with multiple resistance-carrying GIs, creates a high-recombination environment that maximizes horizontal gene transfer potential.

### 4.3. Phylogenetic Context and Host Adaptation

This combination of neurotropic virulence and multidrug resistance in a single strain would conventionally be expected within a phylogroup B2 or D background. Strikingly, despite its high virulence and multidrug resistance, BN01 exhibits the highest genomic relatedness to the commensal laboratory strain *E. coli* K-12 MG1655 (ANI 99.57%) among all compared strains. Phylogenetic analysis positions BN01 with MG1655 as sister to a clade comprising the human UPEC isolate UMN026 and the porcine ExPEC strain PCN033, indicating a shared evolutionary history among ExPEC populations colonizing distinct mammalian hosts. This close relationship between a commensal laboratory strain and a virulent clinical isolate underscores the potential for rapid virulence evolution through horizontal gene transfer.

BN01 belongs to the phylogroup A-ST10-O101:H9 sublineage, recently identified as an emerging clone among bovine ExPEC in China [10]. Unlike classical ExPEC that typically originate from intrinsically pathogenic phylogroups B2 or D, this phylogroup A background represents an alternative evolutionary trajectory: the acquisition of virulence and multidrug resistance traits via plasmids and genomic islands within a commensal lineage. The absence of *cdiA* from the six other representative ExPEC genomes analyzed in this study (see Section 4.1) further highlights the genetic distinctiveness of this bovine-associated sub-lineage. The phylogenetic position of BN01 suggests that bovine-derived ExPEC constitutes a genetically distinct sub-lineage with distinctive ecological adaptations and host-specific epidemiological characteristics. This challenges the conventional phylogroup-virulence association, suggesting that acquired mobile elements can override phylogenetic background in shaping pathogenic potential.

### 4.4. One Health Implications, Zoonotic Risk, and Study Limitations

The findings of this study have important implications under the One Health framework. BN01 represents a bovine ExPEC strain that combines high virulence, extensive antimicrobial resistance, and a complex, complementary plasmid repertoire. The convergence of these traits in a phylogroup A commensal lineage—rather than a classical B2/D ExPEC background—highlights the evolutionary plasticity of livestock-associated *E. coli* and the potential for rapid emergence of pathogenic clones from non-pathogenic ancestors.

From a zoonotic risk perspective, the high nucleotide identity (>95%) of Plas1 (IncI1 to plasmids from human clinical isolates across multiple countries, together with its autonomous conjugative machinery, suggests that this ESBL-carrying plasmid lineage has the capacity for cross-host and transboundary dissemination [28,29].

The unique ~30 kb resistance island on Plas2—absent from the closest characterized relatives in human and swine sources—represents a bovine-specific multidrug resistance configuration, suggesting host-associated evolution of resistance driven by cattle farming practices. Plas3, though resistance-negative, retains complete conjugative machinery and could potentially acquire and disseminate resistance determinants from the bovine microbiome or environment under antimicrobial selection pressure.

Limitations should be acknowledged. This study assessed pathogenicity using a murine meningitis model with intraperitoneal injection; future investigations will employ oral or other natural exposure routes in cattle to better characterize the pathogenicity of BN01 under authentic infection conditions. While genomic analysis provides strong evidence for the horizontal transfer potential of the three plasmids and the GIs, conjugative transfer experiments were not performed to confirm their mobility under laboratory conditions. Additionally, the functional role of the unique *cdiA* gene in BN01 pathogenesis remains to be experimentally validated. Future studies should focus on characterizing the in vivo contribution of *cdiA* to virulence and assessing the transfer efficiency of the three plasmids across clinically relevant bacterial species.

As livestock production intensifies globally, the risk of zoonotic transmission of ExPEC strains from cattle to humans—via direct contact or contaminated food—cannot be overlooked. These findings highlight the need for integrated genomic surveillance bridging human, veterinary, and environmental monitoring, which will be critical for detecting emerging resistance threats and informing evidence-based interventions under the One Health framework.

## 5. Conclusions

This study reports the first complete genome sequence of a bovine ExPEC strain associated with meningoencephalitis, BN01 (O101:H9-ST10-phylogroup A). Unlike classical ExPEC, BN01 represents the emerging A-ST10-O101 sublineage, carrying a distinctive virulence repertoire including *cdiA* and three functionally complementary plasmids: an ESBL carrier (IncI1), a host-specific multidrug resistance island (IncY), and a mobilization-ready vector (IncFII). These features explain its high virulence and extensive multidrug resistance.

This genomic resource reveals that high-risk ExPEC can emerge from phylogroup A—a background traditionally considered commensal. For livestock health, this underscores the need for targeted surveillance and antimicrobial stewardship in cattle populations. From a public health perspective, the cross-species transfer potential of these mobilization-ready vectors highlights the risk of resistance gene dissemination to human pathogens, warranting integrated monitoring under the One Health framework. As the first complete genome for this emerging bovine lineage, these findings establish a foundational reference for understanding ExPEC host adaptation and cross-species transmission potential.

## Figures and Tables

**Figure 1:**
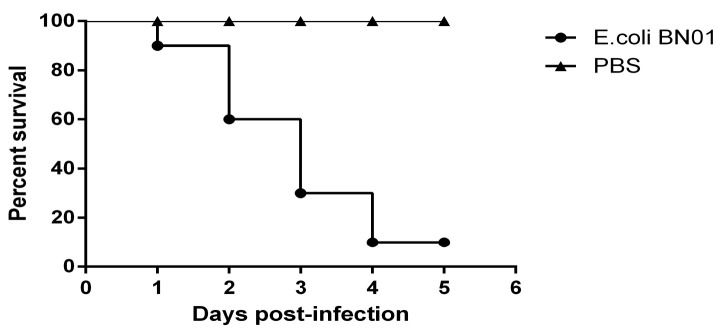
Survival rates of mice following intraperitoneal challenge with *E. coli* BN01.

**Figure 2:**
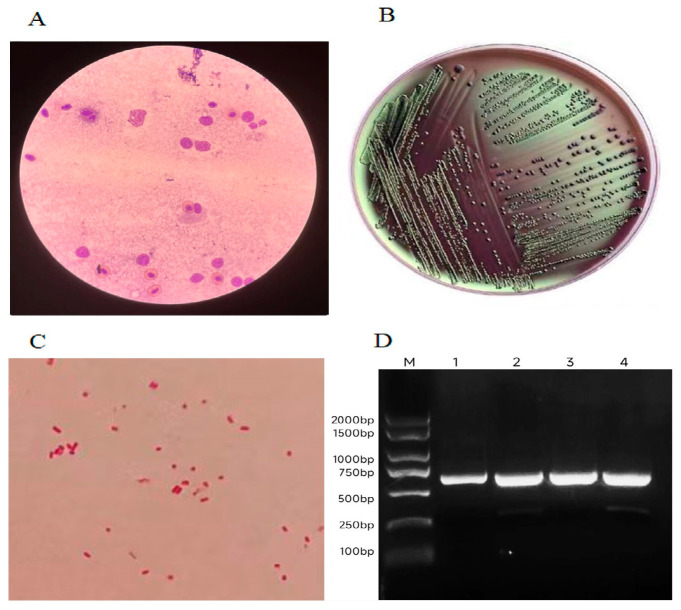
Isolation and identification of bacteria from mouse brain tissue. (**A**) Wright’s staining of brain tissue (×1000); (**B**) eosin methylene blue agar; (**C**) Gram staining microscope (×1000); (**D**) 16S rDNA-based identification of *E. coli*.

**Figure 3:**
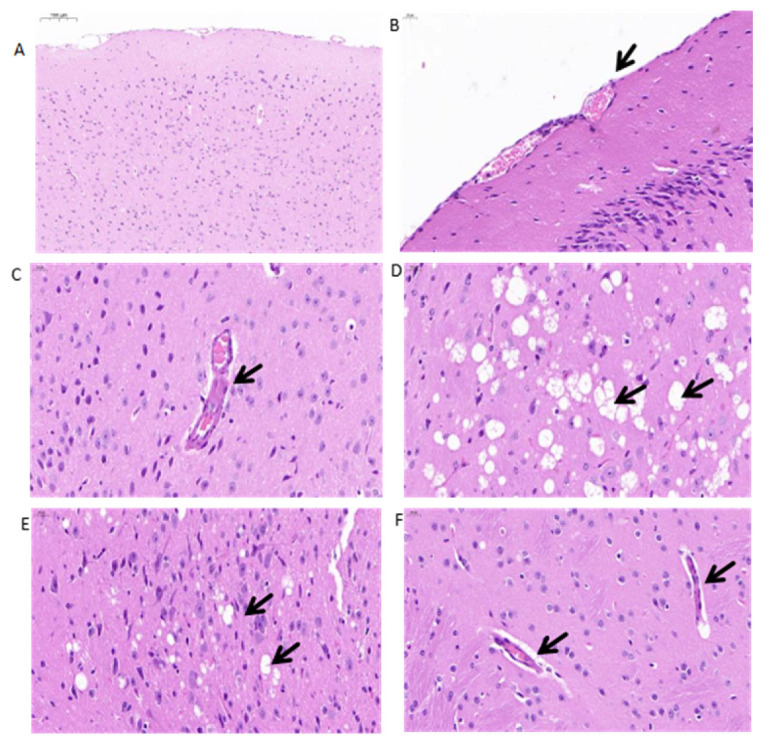
Pathological alterations in brain tissue observed by H&E staining. (**A**) Normal brain architecture without pathological changes (blank control group; 10×); (**B**) submeningeal vascular dilation and congestion (arrow; 40×); (**C**) parenchymal vascular congestion with microthrombi (arrow; 40×); (**D**) diffuse vacuolation of varying sizes in the interstitium (arrow; 40×); (**E**) intracytoplasmic vacuoles within cells (arrow; 40×); (**F**) widened perivascular space (arrow; 40×). Scale bars: 100 μm (**A**) and 20 μm (**B**–**D**).

**Figure 4:**
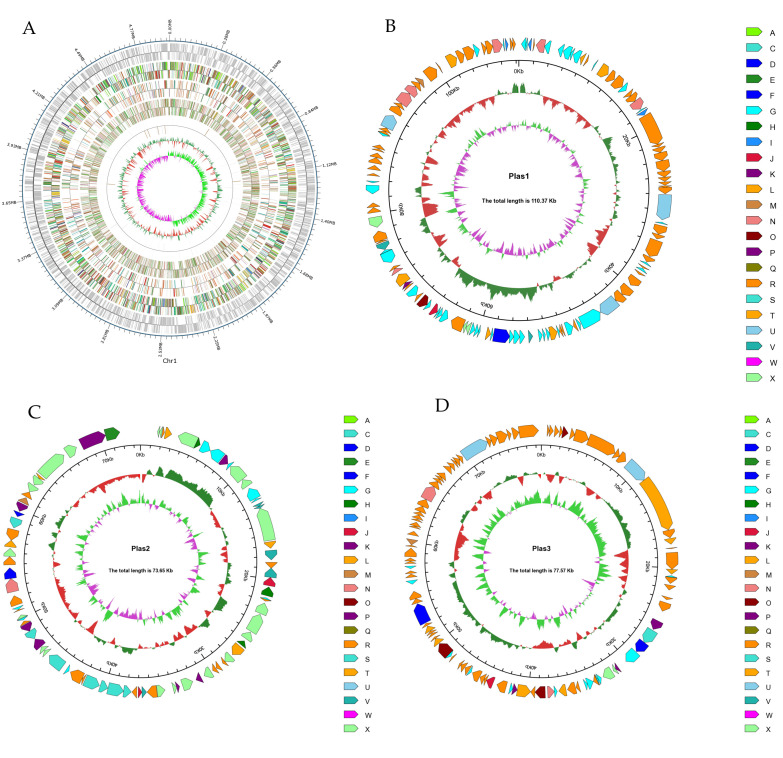
Genomic circular diagrams of *E. coli* BN01. (**A**) Chromosomal map displaying (from outer to inner rings): sequence coordinates, protein-coding genes, functional annotations, ncRNAs, GC content, and GC skew distribution; (**B**–**D**) corresponding plasmid maps (Plas1, Plas2, and Plas3). Note: From the outside to the inside, the images are COG functional annotation classification genes (arrows indicate positive-chain coding clockwise), genomic sequence position coordinates, genomic GC content, and genomic GC skew value distribution.

**Figure 5:**
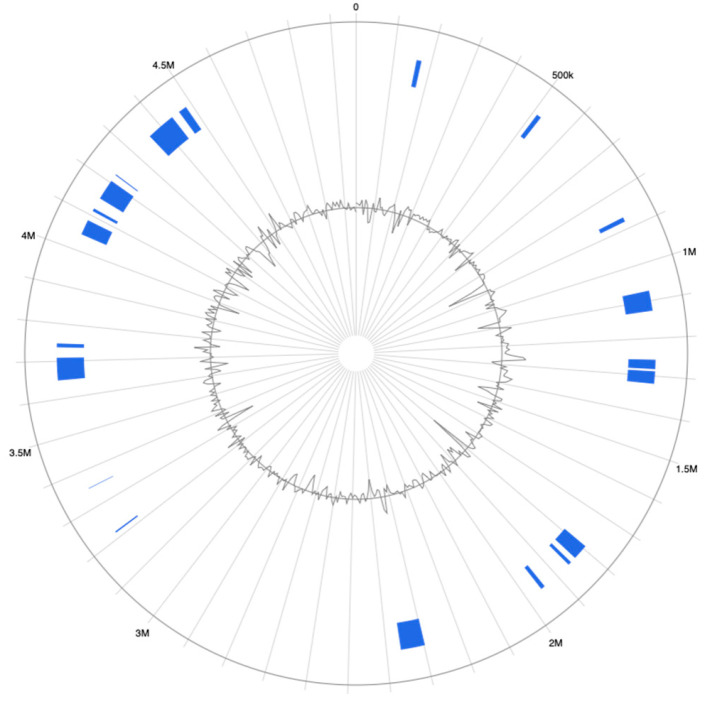
Distribution map of the genomic islands in the BN01 genome.

**Figure 6:**
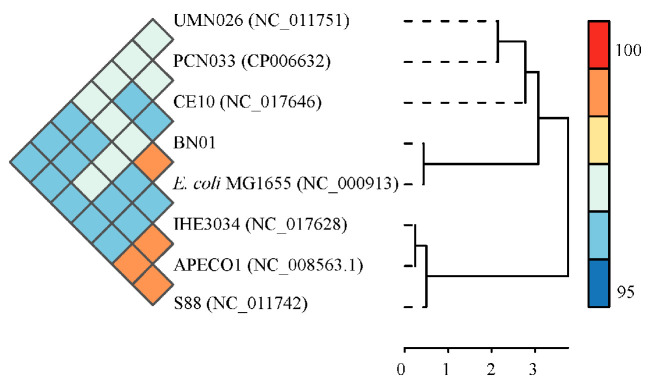
Pairwise ANI heatmap and hierarchical clustering of bovine-derived strain BN01 with seven reference ExPEC strains. Color scale represents ANI values from 95% (blue) to 100% (red).

**Figure 7:**
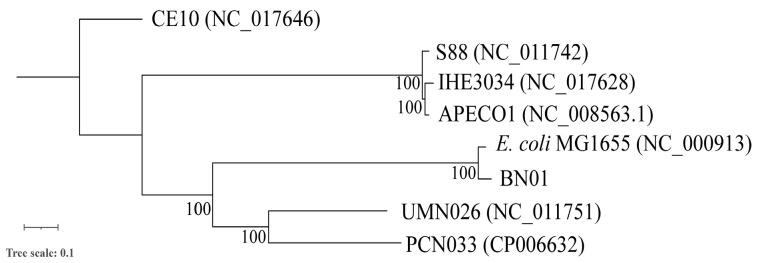
Core-genome phylogenetic tree of BN01 and seven reference ExPEC strains.

**Figure 8:**
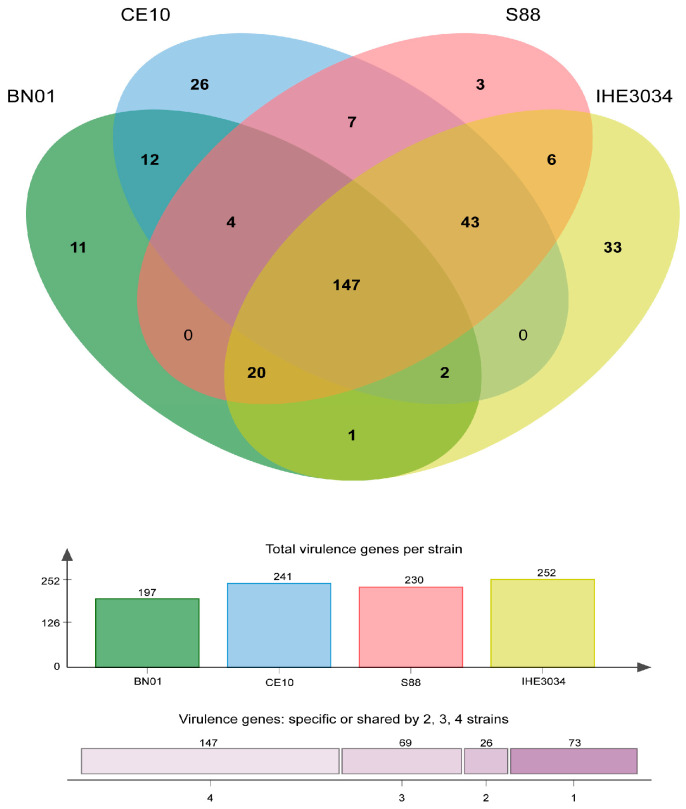
Venn diagram illustrating the distribution of unique and shared virulence genes among the *E. coli* strains BNO1, CE10, S88, and IHE3034: numbers in the overlapping and non-overlapping areas indicate the count of virulence genes shared by multiple strains or unique to a single strain, respectively.

**Figure 9:**
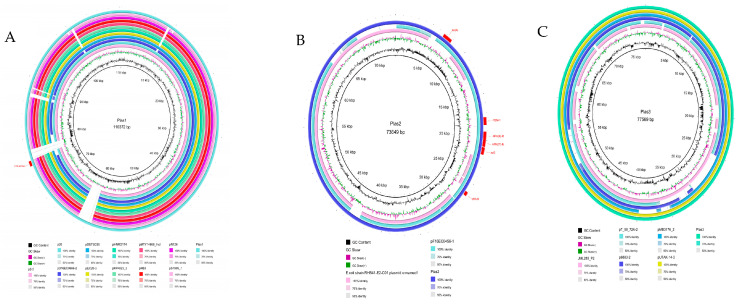
The comparison of Plas1, Plas2 and Plas3 with their respective homologous counterparts. (**A**) The rings, from inside to outside, represent GC content, GC skew, p2-3 (accession no. CP091573.1), p20 (accession no. CP099778.1), pD16EC0666-2 (accession no. CP088595.1), pDETEC89 (accession no. CP116200.1), pEC20-3 (accession no. CP060904.1), pHNRD174 (accession no. KX246268.1), pKP4823_3 (accession no. CP082793.1), pMTY14668_IncI (accession no. CP135648.1), pN83 (accession no. LC567063.1), pN126 (accession no. LC567065.1), pS1905_1 (accession no. CP103772.1), Plas1 and resistance genes. (**B**) The rings, from inside to outside, represent GC content, GC skew, pF16EC0456-1 (accession no. CP088401.1), *E. coli* RHB41-E2-C01 plasmid unnamed1 (accession no. CP099107.1), Plas2 and resistance genes. (**C**) The rings, from inside to outside, represent GC content, GC skew, JML285_P2 (accession no. AP024116.1), p7_58_72A-2 (accession no. CP077501.1), p6883-2 (accession no. CP075000.1), pMB3176_2 (accession no. CP103720.1), pUTAK-14-3 (accession no. CP169001.1), Plas3.

**Figure 10:**
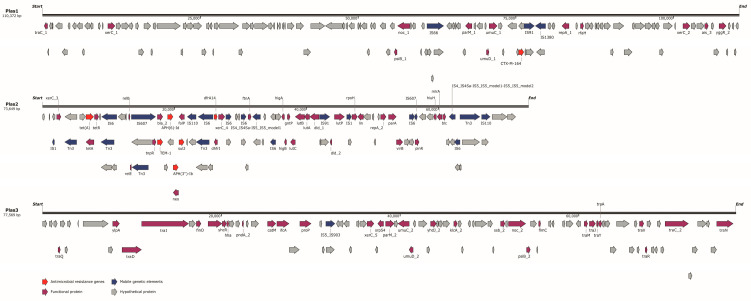
The genetic structures of antimicrobial resistance genes (ARGs) on plasmids. The presence of ARGs is represented by red arrows, mobile genetic elements are indicated by blue arrows, functional protein genes are denoted by pink arrows, and hypothetical protein genes are represented by gray arrows.

**Table 1:** Calculation of LD_50_ of SPF mice infected with *E. coli* BN01.

Strain	Group	Does CFU/mL	No. of Animals	Death No.	Mortality	LD50 CFU/Mouse
BN01	1	5.3 × 10^9^	10	10	1	10^6.3^
	2	5.3 × 10^8^	10	8	0.80	
	3	5.3 × 10^7^	10	8	0.80	
	4	5.3 × 10^6^	10	3	0.30	
	5	5.3 × 10^5^	10	3	0.30	
PBS	1	—	10	0	0	—

—: No data.

**Table 2:** Overall genome features of *E. coli* BN01.

Characteristic	*E. coli* BN01
Genome size (bp)	5,269,374
G+C content (%)	50.82
Contings	4
Average gene size (bp)	903
rRNA	22
tRNA	87
sRNA	63
Genomic island (GIs)	20
prophage	10
CRISPR	36
Serotype	O101:H9

**Table 3:** Genomic features and pairwise ANI values between the BN01 strain and selected *E. coli* isolates.

Strain	Host	Pathotype	Serotype	Phylogroup	ST	ANI Value with BN01
BN01	bovine	ExPEC (MNEC)	O101:H9	A	ST10	-
CE10	human	MNEC	O7:K1	F	ST62	97.10
S88	human	MNEC	O45:K1:H7	B2	ST95	96.67
IHE3034	human	MNEC	O18:K1:H7	B2	ST95	96.63
UMN026	human	UPEC	O7:K1	D	ST69	97.22
APECO1	poultry	APEC	O1:K1:H7	B2	ST95	96.62
PCN033	porcine	ExPEC	O11	D	ST131	97.92
MG1655		Commensal	OR:H48:K	A	-	99.57

**Table 4:** Analysis results of *E. coli* BN01 drug resistance genes.

Resistance Mechanism	Antibiotic Resistant Genes (Number)
Antibiotic inactivation	*aph(3′’)-Ib*,*aph(6)-Id*, *aav(3)-IId*, *aac(6′)-Ib7*, *aac(6′)-Ib3*, *blcCTX-M-14*, *blcCTX-M-164*, *blc*CTX-M-55, *blc*TEM-1B, *ampH*, *ampC*, *mphA*, *mphB*, *fosA3*
Reduced permeability to antibiotic	*marA, cmlA5, cmlA1*
Antibiotic target alteration	*bacA*, *sul2*, *dfrA14*, *gyrA*, *parC*, *parE*, *eptA*, *pmrF*
ATP-binding cassette (ABC) antibiotic efflux pump; major facilitator superfamily (MFS) antibiotic efflux pump; resistance-nodulation-cell division (RND) antibiotic efflux pump	*Pseudomonas aeruginosa soxR*, *tolC*
ATP-binding cassette (ABC) antibiotic efflux pump	*yojI*, *msbA*
Major facilitator superfamily (MFS) antibiotic efflux pump	*emrB*, *emrA*, *emrR*, *emrK*, *emrY*, *mdtH*, *mdtG*, *mdtN*, *mdtO*, *mdtP*, *mdtM*, *mdfA*, *floR*, *tet(A)*
resistance-nodulation-cell division (RND) antibiotic efflux pump	*acrS*, *acrA*, *acrB*, *acrD*, *mdtA*, *mdtB*, *mdtC*, *mdtE*, *mdtF*, *gadX*, *gadW*, *CRP*, *baeR*, *baeS*, *marA*, *cpxA*
Major facilitator superfamily (MFS) antibiotic efflux pump; resistance-nodulation-cell division (RND) antibiotic efflux pump	*evgA*, *evgS*, *H-NS*

## Data Availability

The original contributions presented in this study are included in the article/supplementary material. Further inquiries can be directed to the corresponding authors.

## Supplementary Materials

The following supporting information can be downloaded at https://www.mdpi.com/article/10.3390/microorganisms14071586/s1, Table S1: Information on the Gene islands of *E. coli* BN01; Table S2: Prediction of Virulence Genes in *E. coli* BN01; Table S3: Drug sensitivity test results. Figure S1: Neuropathological features of the natural bovine case infected with BN01; Figures S2–S8: Functional Annotation of the *E. coli* BN01 Genome.
