# Supplementary material for: High-Risk ExPEC from Commensal Phylogroup A: Genomic Characterization of a Bovine Meningoencephalitis Isolate, BN01
Source: Microorganisms. 2026 Jul 21;14(7):1586. doi: 10.3390/microorganisms14071586 (PMC13413666; doi:10.3390/microorganisms14071586)
Supplement: Supplementary file 1 [file microorganisms-14-01586-s001.zip › Figure S1. Neuropathological features of the natural bovine case infected with BN01.pdf]

A one-week-old neonatal calf displaying terminal opisthotonos revealed severe meningeal hemorrhage at necropsy (Figure S1A). Histopathological examination (H&E staining) demonstrated submeningeal vascular congestion, extensive neutrophil infiltration within meninges and perivascular spaces, and eosinophilic fibrillar exudates forming delicate meshworks (Figure S1B). Affected neurons exhibited karyopyknosis with chromatin condensation and cytoplasmic shrinkage, accompanied by markedly widened perivascular spaces (Figure S1C). Classic perivascular cuffing of inflammatory cells with concurrent perivascular space dilation was observed around small vessels (Figure S1D).

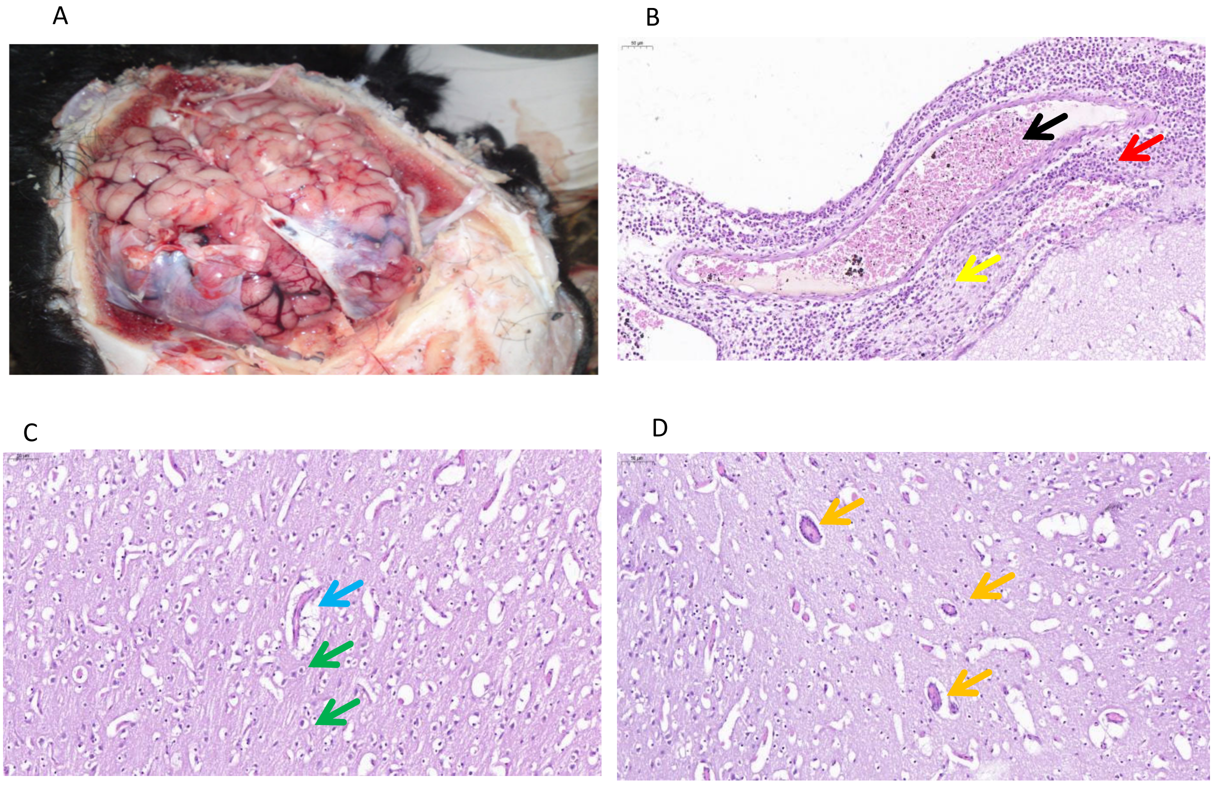

Figure S1: Neuropathological features of the natural bovine case infected with BN01. (A) Meningeal hemorrhage; (B) Submeningeal vascular dilation and congestion (black arrow; 20×), eosinophilic fibrinous exudate (yellow arrow; 20×), neutrophil infiltration (red arrow; 20×); (C) Widening of perivascular space (blue arrow; 20×), affected neurons showing karyopyknosis, condensed chromatin, and shrunken cytoplasm (green arrow; 20×); (D) Perivascular cuffing of inflammatory cells with widening of perivascular space (orange arrow; 20×).
