# Supplementary material for: High-Risk ExPEC from Commensal Phylogroup A: Genomic Characterization of a Bovine Meningoencephalitis Isolate, BN01
Source: Microorganisms. 2026 Jul 21;14(7):1586. doi: 10.3390/microorganisms14071586 (PMC13413666; doi:10.3390/microorganisms14071586)

Figure S2. GO function classification map of the BN01 genome: The abscissa indicates the number of genes on the annotation; the vertical axis indicates the percentage of the number of genes on the annotation to all coding genes.

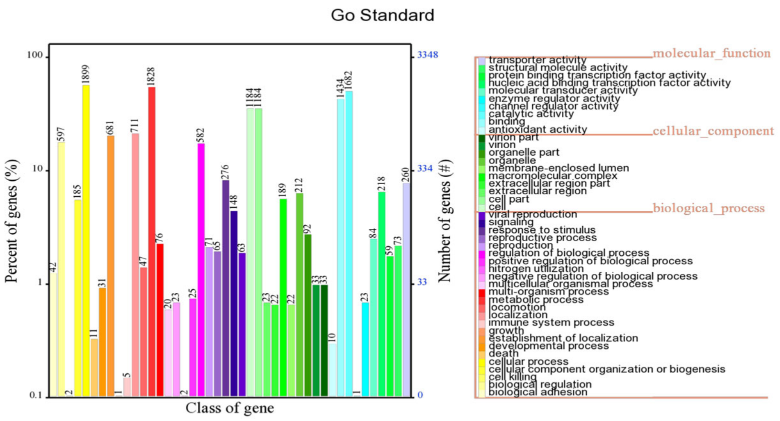

Figure S3. Classification diagram of KEGG metabolic pathway of the BN01 genome: The number on the bar graph represents the number of genes annotated to the KEGG pathway. The ordinate axis is the name of each KEGG pathway in level 1 in the database.

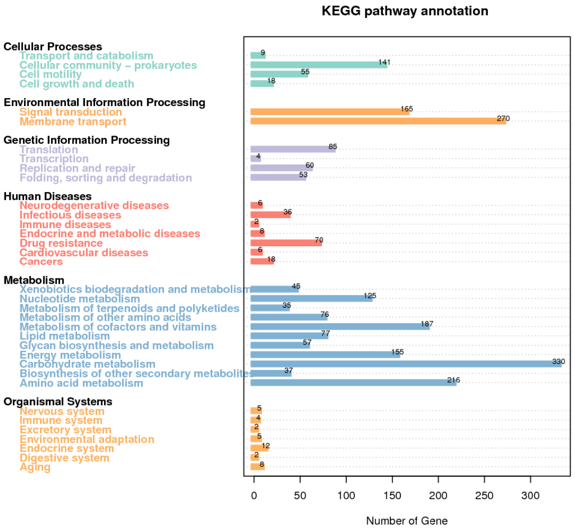

Figure S4. COG function classification diagram of the BN01 genome: the abscissa indicates the type of COG function; the ordinate indicates the number of genes.

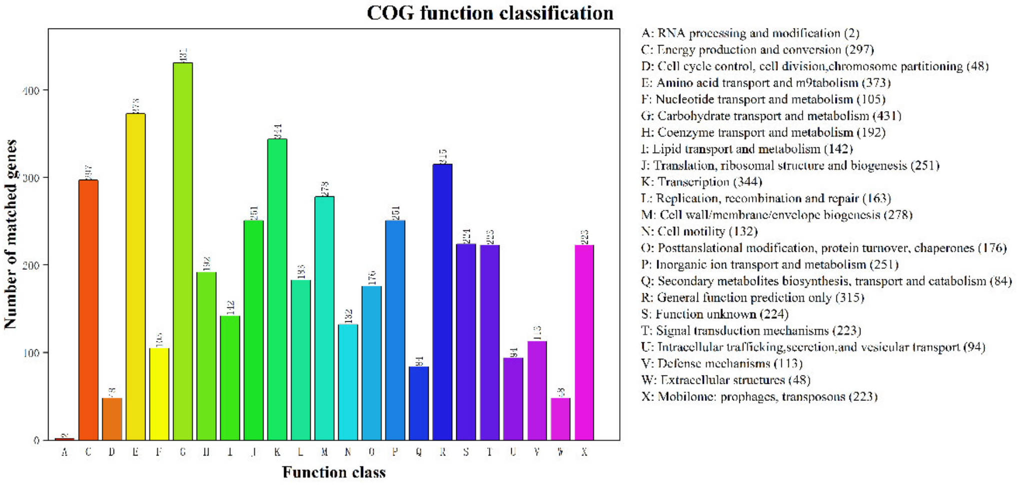

Figure S5. NR Functional Classification Diagram: the abscissa indicates species ID, and the the ordinate indicates the number of genes annotated for each species in the NR.

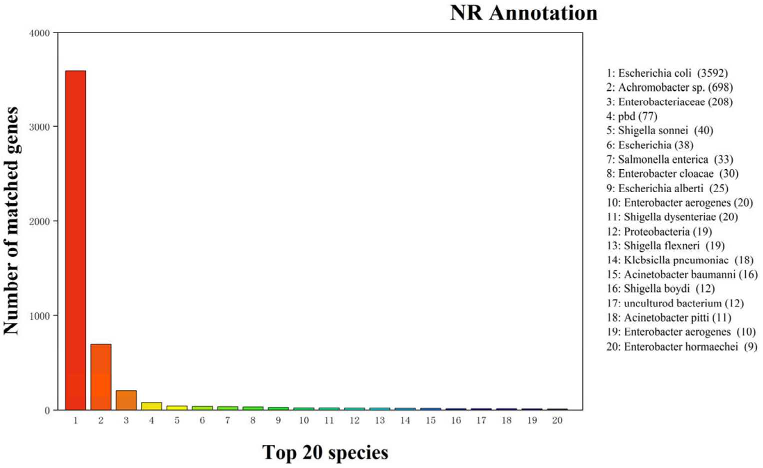

Figure S6. TCDB function classification diagram of BN01 genome: The abscissa indicates the primary classification type of TCDB; The ordinate indicates the number of genes on the annotation.

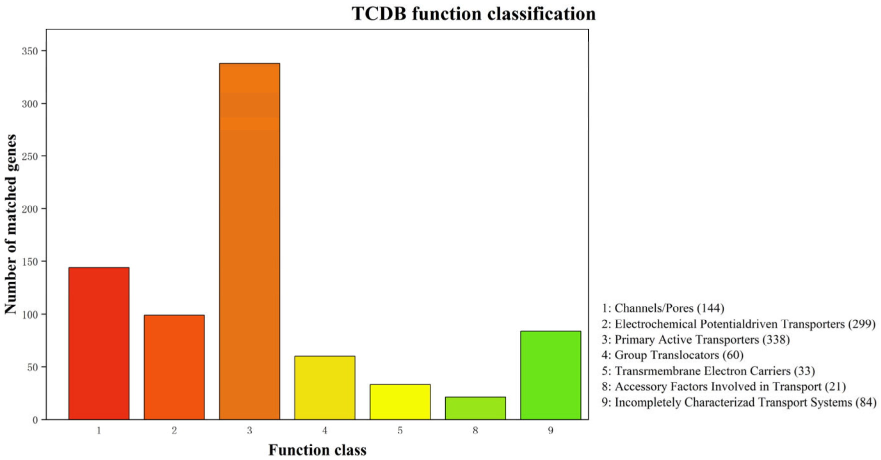

Figure S7. CAZy Functional Classification and Corresponding Gene Quantity Statistical Chart:The abscissa indicates the classification classs of CAZy; The ordinate indicates indicates the number of genes on the annotation to all coding genes.

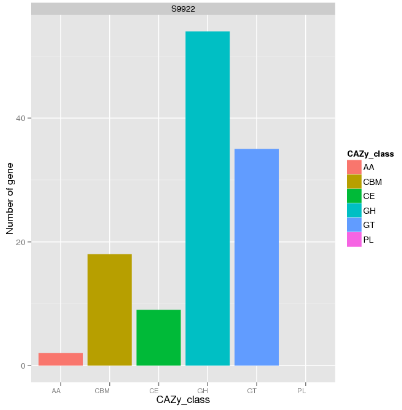

Figure S8. PHI database annotation results of BN01 genome: The abscissa indicates the phenotypic mutation type. The ordinate indicates the number of genes on the annotation.

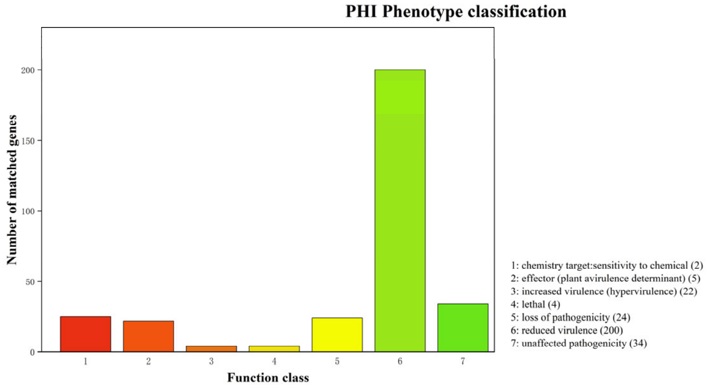

Supplement: Supplementary file 1 [file microorganisms-14-01586-s001.zip › Figures S2-S8. Functional Annotation of the E.coli BN01 Genome.pdf]
