# Supplementary material for: High-Risk ExPEC from Commensal Phylogroup A: Genomic Characterization of a Bovine Meningoencephalitis Isolate, BN01
Source: Microorganisms. 2026 Jul 21;14(7):1586. doi: 10.3390/microorganisms14071586 (PMC13413666; doi:10.3390/microorganisms14071586)
Supplement: Supplementary file 1 [file microorganisms-14-01586-s001.zip › Table S3. Drug sensitivity test results.pdf]

Table S3. Drug sensitivity test results

Drug type	Drugs	Drug content(μg)	Results
Aminoglycoside	amikacin	30	R
	gentamicin	10	R
	tobramycin	10	R
β-lactams	ampicillin	10	R
	cefotaxime	30	R
	cefazolin	30	R
	ceftazidime	30	R
	amoxicillin-clavulanate	20/10	R
Tetracyclines	doxycycline	30	S
	minocycline	30	S
	tetracyclinea	30	R
Chloramphenicols	chloramphenicol	30	R
Quinolones	ciprofloxacin	5	R
Carbapenem	imipenem	10	I
	ertapenem	10	S
Sulfonamides	co-trimoxazole	1.25/23.75 μg	R

R,Resistant, S, Sensitive, I, Intermediary
